# Prices and availability of locally produced and imported medicines in Ethiopia and Tanzania

**DOI:** 10.1186/s40545-016-0095-1

**Published:** 2017-01-16

**Authors:** M. Ewen, W. Kaplan, T. Gedif, M. Justin-Temu, C. Vialle-Valentin, Z. Mirza, B. Regeer, M. Zweekhorst, R. Laing

**Affiliations:** 1 Health Action International (HAI), Overtoom 60/II, 1054HK, Amsterdam, Netherlands; 2 Boston University School of Public Health, Boston, MA USA; 3 University of Western Cape, Cape Town, South Africa; 4 University of Addis Ababa, Addis Ababa, Ethiopia; 5 Muhimbili University of Health and Allied Sciences, Dar Es Salaam, Tanzania; 6 Harvard Pilgrim Health Care Institute, Boston, MA USA; 7 World Health Organization Regional Office for the Eastern Mediterranean, Cairo, Egypt; 8 Athena Institute, VU University, Amsterdam, Netherlands

**Keywords:** Medicines, Locally produced, Imported, Prices, Availability

## Abstract

**Background:**

To assess the effect of policies supporting local medicine production to improve access to medicines.

**Methods:**

We adapted the WHO/HAI instruments measuring medicines availability and prices to differentiate local from imported products, then pilot tested in Ethiopia and Tanzania. In each outlet, prices were recorded for all products in stock for medicines on a country-specific list. Government procurement prices were also collected. Prices were compared to an international reference and expressed as median price ratios (MPR).

**Results:**

The Ethiopian government paid more for local products (median MPR = 1.20) than for imports (median MPR = 0.84). Eight of nine medicines procured as both local and imported products were cheaper when imported. Availability was better for local products compared to imports, in the public (48% vs. 19%, respectively) and private (54% vs. 35%, respectively) sectors. Patient prices were lower for imports in the public sector (median MPR = 1.18[imported] vs. 1.44[local]) and higher in the private sector (median MPR = 5.42[imported] vs. 1.85[local]). In the public sector, patients paid 17% and 53% more than the government procurement price for local and imported products, respectively.

The Tanzanian government paid less for local products (median MPR = 0.69) than imports (median MPR = 1.34). In the public sector, availability of local and imported products was 21% and 32% respectively, with patients paying slightly more for local products (median MPR = 1.35[imported] vs. 1.44[local]). In the private sector, local products were less available (21%) than imports (70%) but prices were similar (median MPR = 2.29[imported] vs. 2.27[local]). In the public sector, patients paid 135% and 65% more than the government procurement price for local and imported products, respectively.

**Conclusions:**

Our results show how local production can affect availability and prices, and how it can be influenced by preferential purchasing and mark-ups in the public sector. Governments need to evaluate the impact of local production policies, and adjust policies to protect patients from paying more for local products.

## Background

Ensuring access to medicines requires policies to improve the availability and affordability of quality-assured medicines that meet local health needs [1]. Surveys using the World Health Organization (WHO)/Health Action International (HAI) tool measuring medicine prices, availability and affordability [2] have shown poor medicine availability (particularly in the public sector), high prices in both the public and private sectors, and unaffordable treatments for those on low wages [3–7]. Use of the WHO/HAI survey tool has greatly increased knowledge on medicine prices and availability in low- and middle-income countries (LMICs), but the analysis does not differentiate between local and imported products.

Increasingly, governments in LMICs are supporting local medicine production, expecting that it will result in increased availability and lower prices, as well as industrial and economic benefits [8]. To assist countries the WHO, in partnership with the United Nations Conference on Trade and Development (UNCTAD) and the International Centre for Trade and Sustainable Development (ICTSD), commenced a project in 2012 on local production of medical products for improved access in LMICs. A literature review in Part 1 of the project found inconclusive evidence as to whether local production improved access to medicines [9]. The authors proposed that to shed light on this issue, surveys using the WHO/HAI survey tool should differentiate between local and imported medicines [10]. Therefore, in Part 2 of the project, such a tool was developed and pilot tested.

### Objective

The objective of this study was to compare the price and availability of locally produced and imported medicines and, in particular, to answer the following key questions:

- What prices does the government pay, and quantities procured, for selected medicines that are locally produced and imported, and how do these prices compared with public sector patient prices?
- What is the availability and patient price for locally produced and imported medicines in different sectors and regions of the country?
- Do prices and availability vary by product type (originator brands, branded generics and International Non-proprietary Name (INN) generics)?
- Do prices vary by country of manufacture?
- How do prices compare with international reference prices?

The WHO/HAI survey tool was adapted to measure the price and availability of locally produced and imported medicines, and answer the study questions. In August 2013 the new tool was pilot tested Tanzania and Ethiopia, two of the East African countries in the WHO local production project, with the support of the governments in both countries.

### Pharmaceutical sectors

In Ethiopia there were nine local pharmaceutical manufacturers, while in Tanzania there were seven. All made finished dosage forms, but not active pharmaceutical ingredients. Prices were not regulated in either country, nor were mark-ups regulated in the pharmaceutical supply chain. In Ethiopia, the annual pharmaceutical, supplies and medical equipment budget was 8.26 billion Birr (approximately USD$441 million) in 2013/14. The Pharmaceutical Fund and Supply Agency procures medicines on behalf of the Ethiopian Government via centralised international tenders. In Tanzania, the budget for medicines, medical equipment and building capacity in the health sector was TShs. 92.05 billion (approximately USD$58 million) in 2013/14. The Medical Stores Department procures medicines on behalf of the Tanzanian Government, also via centralised international tenders. Both countries award tenders to the lowest-priced bid submitted by prequalified suppliers, except local manufacturers are permitted a preference of up to 25% in Ethiopia and 15% in Tanzania. In both countries, patients pay for most medicines out-of-pocket in the public sector (some medicines in the Tanzanian public and mission sectors are provided as part of the consultation fee). Neither country taxes medicines, but Ethiopia applies a 5% import tariff on finished products.

## Method

### Study design

#### Sampling

In each country, patient price and availability data were collected in the capital and five other regions, as per the WHO/HAI methodology. In Ethiopia, the survey areas were Addis Ababa, Oromia, Amhara, Southern Nations, Nationalities, and Peoples’ Region (SNNPR), Harari and Afar. In Tanzania, data were collected in Dar es Salaam, Manyara, Mbeya, Mtwara, Shinyanga and Tabora.

This article reports on the findings from the public and private sectors. In Ethiopia, 34 public sector outlets were sampled (hospital pharmacies and health facilities) and 30 private retail pharmacies. In Tanzania, data were collected from 33 public sector outlets (hospital pharmacies and health facilities) and 30 private sector outlets (private retail pharmacies, and Accredited Drug Dispensing Outlets which are privately-owned outlets licensed to sell certain essential medicines). Data were also collected from a third sector (mission facilities in Tanzania, and NGO and municipal pharmacies in Ethiopia) but are not included here. The data for these sectors can be accessed in the two country reports on HAI’s website (http://haiweb.org/what-we-do/price-availability-affordability/measuring-the-availability-and-prices-of-locally-produced-and-imported-medicines/).

Current government procurement prices and quantities were also collected. In Ethiopia, these were 2013 tender prices collected from the Pharmaceutical Fund and Supply Agency. In Tanzania, 2012 tender prices were collected from the Medical Stores Department.

#### Medicines

Data were collected and analysed for 25 medicines in Ethiopia, and 24 in Tanzania (Table 1). The medicines were selected nationally, were strength- and dosage form-specific, and were made by at least one local manufacturer. In each outlet, for each medicine data were collected on all products in stock with the same active ingredient(s), strength and dosage form. The country of manufacture was identified from product labels.

**Table 1:** Survey medicines

Ethiopia	Tanzania
Acetyl salicyclic acid 300 mg tab/cap	Acetyl salicyclic acid 300 mg tab/cap
Albendazole 100 mg/5 ml suspension	Albendazole 100 mg/5 ml suspension
Amoxicillin 250 mg tab/cap	Amoxicillin 250 mg tab/cap
Amoxicillin 500 mg tab/cap	Amoxicillin 500 mg tab/cap
Chloramphenicol 250 mg tab/cap	Chloramphenicol 250 mg tab/cap
Ciprofloxacin 500 mg tab/cap	Ciprofloxacin 500 mg tab/cap
Diclofenac 50 mg tab/cap	Diclofenac 50 mg tab/cap
Doxycyline 100 mg tab/cap	Doxycyline 100 mg tab/cap
Erythromycin 250 mg tab/cap	Erythromycin 250 mg tab/cap
Paracetamol 120 mg/5 ml suspension	Paracetamol 120 mg/5 ml suspension
Paracetamol 500 mg tab/cap	Paracetamol 500 mg tab/cap
Sulfamethoxazole + Trimethoprim 400 mg + 80 mg tab/cap	Sulfamethoxazole + Trimethoprim 400 mg + 80 mg tab/cap
Tetracycline 250 mg tab/cap	Tetracycline 250 mg tab/cap
Amitriptyline 25 mg tab/cap	Artemeter + Lumefantrine 20 mg + 120 mg tab/cap
Benzathine penicillin 2.4MIU injection	Azithromycin 250 mg tab/cap
Chloroquine 50 mg/5 ml syrup	Cloxacillin 250 mg tab/cap
Enalapril 10 mg tab/cap	Erythromycin 125 mg/5 ml suspension
Fluoxetine 20 mg tab/cap	Fluconazole 150 mg tab/cap
Furosemide 40 mg tab/cap	Ibuprofen 200 mg tab/cap
Glibenclamide 5 mg tab/cap	Quinine sulphate 300 mg tab/cap
Ibuprofen 400 mg tab/cap	Salbutamol 4 mg tab/cap
Metoclopramide 5 mg/5 ml syrup	Sulfadoxine + Pyrimethamine 500 mg + 25 mg tab/cap
Metronidazole 250 mg tab/cap	Sulfamethoxazole + Trimethoprim 200 + 40 mg/5 ml suspension
Phenobarbitone 100 mg tab/cap	Zinc sulphate 20 mg dispersible tab
Sodium Chloride 0.9% 1 L IV solution	

### Data quality assurance

National investigators were trained in a two-day workshop which included piloting data collection. They then trained their survey personnel. Prices were identified from packs or pharmacy computers. Data were checked at the end of each day for completeness and possible errors. Re-surveying three outlets per country did not reveal any inconsistencies in the data collected. Data were double-entered into the automated Excel Workbook. The country of manufacture and marketing authorization was validated with the Tanzanian Food and Drug Administration (TFDA), and checked on the website of the Ethiopian Food, Medicine and Health Care Administration and Control Authority (FMHACA).

### Data analysis

In this study, local production was defined as products that were manufactured and packaged/labelled in the study country.

Availability was based on whether the medicine was in stock on the day of data collection at the surveyed facility. All medicines were included in the availability analysis.

International Commercial (INCO) terms were identified for each product procured by the government. To be more comparable with prices of locally produced products, adjustments were made to prices of imports which did not cover all costs to the national government store.

For each medicine, where more than one locally produced or imported product was found in an outlet, the median unit price was used in the analysis.

Prices were expressed as median price ratios (MPR). An MPR is the ratio of the price in local currency (Tanzanian Shilling/Ethiopian Birr) divided by an international reference price (IRP) converted to local currency using the exchange rate on the first day of data collection. The MPR is thus an expression of how much greater or less the price in the country is than the IRP e.g. an MPR of 1 means the country price was equivalent to the IRP, whereas an MPR of 2 means the country price was twice that of the IRP. In these surveys, use of IRPs serve as a benchmark for price comparisons between locally produced and imported medicines. The IRPs were taken from the 2012 Management Sciences for Health International Drug Price Indicator Guide (current at the time of the surveys) for international procurements. They reflect prices that governments in LMICs could be expected to pay for medicines. According to WHO and HAI, governments in LMICs should be able to achieve an MPR of 1 when buying medicines, and WHO considers patient prices are high when MPRs exceed 4 [11].

For patient prices, an MPR was only calculated for a medicine when at least four price points were recorded per sector. For public procurement prices, an MPR was calculated when one or more prices were recorded. Most analyses in this article are paired i.e. the analysis includes only medicines (same strength and dosage forms) where MPRs were calculated for both local and imported products.

Prices and availability of all products were also analysed by product type i.e. originator brands, branded generics and International Non-proprietary Name (INN) generics. An originator brand is the product that was first authorized world-wide for marketing (usually as a patented product) and always has a brand name. A branded generic is a generic equivalent product marketed under a brand name. An INN generic is a generic equivalent product marketed under its INN name.

## Results

### Ethiopia

#### Government procurement prices and quantities

Of the 25 survey medicines, the government procured 21 locally produced medicines (48 products) and 12 imported medicines (13 products). Based on the INCO terms, 22% was added to the procurement price of nine products found to be Free Carrier or Free on Board (15% freight, 0.5% insurance, 1.5% bank charges, 5% import duty) and 7% was added to four products found to be Cost and Freight (0.5% insurance, 1.5% bank charges, 5% import duty).

Overall, government procurement prices for locally produced and imported medicines were 1.20 and 0.84 times international reference prices (IRP), respectively (Table 2). For local products, half ranged from 0.99-1.33 times IRPs, whereas for imported products half were 0.77-1.26 times IRPs.

**Table 2:** Summary of government procurement prices, availability and patient prices in Ethiopia for locally produced and imported medicines

		Public sector		Private sector	
		Locally produced	Imported	Locally produced	Imported
Government procurement prices^a^	Number of medicines (products)	21 (48)	12 (13)	N/A	
	Median MPR	1.20	0.84		
	Interquartile range	0.99 – 1.33	0.77 – 1.26		
Availability	Mean availability of all products (local and imported) and product types	64%		73%	
	Mean availability of all product types -Originator brands -Branded generics -INN generics	48% 0% 37% 14%	19% 0% 10% 9%	54% 0% 42% 13%	35% 9% 29% 3%
Patient prices	Number of medicines (products)	10 (177)	10 (129)	15 (306)	15 (403)
	Median MPR	1.44	1.18	1.85	5.42
	Median interquartile range	1.08-1.56	1.02-1.42	1.71-1.96	2.65-9.34
	Median MPR (products)^a^ -Originator brands -Branded generics -INN generics	ᅟ - 1.41 (331) 1.45 (125)	ᅟ - 1.14 (89) 1.41 (79)	ᅟ - 1.71 (330) 2.17 (98)	ᅟ 20.35 (69) 4.33 (411) 2.08 (25)

^a^Unpaired analysis of prices

For nine medicines the government procured both local and imported products, at variable prices and quantities. For example, for ciprofloxacin five locally produced products (total of 49.295 million tablets at 0.6580-0.7300 Birr per tablet) and one imported product (13.6 million tablets at 0.5119 Birr per tablet) were purchased. The lower priced imported product accounted for only 21.6% of the total quantity of ciprofloxacin purchased. For eight of the nine medicines, median procurement prices of local products were higher (45% more) than those of imported products. They ranged from 1% more for doxycycline to 134% more for erythromycin. The sole exception was locally produced phenobarbitone which was a third of the price of the imported product. For these eight medicines, the government would have saved about $3.7 million USD in 2013 if only the imported products were procured.

#### Availability and patient prices in the public sector

The mean availability of the medicines (whether imported or locally produced) in the public sector outlets was 64% (Table 2). Local products had greater mean availability (48%) than imported products (19%). The availability for individual medicines was highly variable. Branded generics (37%) were more commonly found than INN generics (14%) for local products, whereas for imported products the availability of branded generics (10%) was similar to INN generics (9%). No originator brands were found in the public sector.

Public sector patient prices for local products were higher priced (median MPR = 1.44) than imported products (median MPR = 1.18) across the 10 medicines in the paired analysis. Hence, patients were paying 22% more when being dispensed local products.

Across all medicines (unpaired), patients in the public sector were paying 23% more for locally produced branded generics (median MPR = 1.41) than imports (median MPR = 1.14). For INN generics, the difference was minimal.

#### Public sector patient prices compared to government procurement prices

For the 20 locally produced medicines that the government procured and sold to patients in public sector outlets (paired analysis), patients were paying an average of 1.17 times (17% more than) the government procurement price (Table 3). For imported products (9 medicines), patients were paying 1.53 times (53% more than) the procurement price.

**Table 3:** Median ratio between public sector patient prices and procurement prices for locally produced and imported medicines

	Number of paired medicines	Median ratio between public sector patient price MPR and public sector procurement price MPR
Ethiopia		
Locally produced products	20	1.17
Imported products	9	1.53
Tanzania		
Locally produced products	8	2.35
Imported products	7	1.65

#### Availability and patient prices in the private sector

Mean availability of the medicines (imported or locally produced) was 73% in the private sector (Table 3). Availability of local and imported products was 54% and 35%, respectively, with variability for individual medicines. Branded generics were more commonly found than INN generics for local products (42% vs 13%) and imported products (29% vs 3%). No locally produced originator brands were found. The mean availability of imported originator brands was 9%.

Overall, patient prices for local products (median MPR = 1.85) were lower than imported products (median MPR = 5.42) across the 15 medicines in the paired analysis (Table 3). Overall, patients were paying 193% more for imported products in the private sector.

Across all medicines (unpaired), imported branded generics (median MPR = 4.33) were 153% higher priced than local branded generics (median MPR = 1.71). Locally produced INN generics were slightly higher priced (median MPR = 2.17) than imported INN generics (median MPR = 2.08). The few imported originator brands were far high priced (median MPR = 20.35) compared to the generics.

### Tanzania

#### Government procurement prices and quantities

For each medicine procured by the government, locally produced or imported products were purchased but not both. Of the 24 survey medicines, the government procured 9 locally produced medicines (9 products) and 7 imported medicines (10 products). Based on the INCO terms, no price adjustments were needed. Overall, government procurement prices for local and imported products were 0.69 and 1.34 times IRPs, respectively (Table 4). For local products, half ranged from 0.65-0.97 times IRPs, whereas for imported products half were 0.69-4.85 times IRPs.

**Table 4:** Summary of government procurement prices, availability and patient prices in Tanzania for locally produced and imported medicines

		Public sector		Private sector	
		Locally produced	Imported	Locally produced	Imported
Government procurement prices^a^	Number of medicines (products)	9 (9)	7 (10)	N/A	
	Median MPR	0.69	1.34		
	Interquartile range	0.65 – 0.97	0.69 –4.85		
Availability	Mean availability of all products (local and imported) and product types	52%		82%	
	Mean availability of all product types -Originator brands -Branded generics -INN generics	21% 0% 15% 6%	32% 4% 27% 5%	21% 0% 19% 2%	70% 7% 58% 12%
Patient prices	Number of medicines (products)	9 (104)	9 (107)	12 (131)	12 (331)
	Median MPR	1.44	1.35	2.27	2.29
	Median interquartile range	1.00-1.83	1.29-1.75	2.07-2.95	2.18-3.14
	Median MPR (products)^a^ -Originator brands -Branded generics -INN generics	ᅟ - 1.67 (121) 1.98 (49)	ᅟ 3.48 (42) 2.20 (230) 1.97 (36)	ᅟ - 2.01 (149) 2.07 (12)	ᅟ 8.79 (50) 2.97 (567) 2.38 (96)

^a^Unpaired analysis of prices

#### Availability and patient prices in the public sector

The mean availability of the medicines (imported or locally produced) in the public sector outlets was 52% (Table 4). Imported products had greater availability (32%) than local products (21%), with variability for individual medicines. Branded generics (15%) were more commonly found than INN generics (6%) for local products. The same was seen for imported medicines; availability of branded and INN generics was 27% and 5%, respectively. Originator brands were rarely available at 4% and 0% for imported and local products, respectively.

Public sector patient prices for local products were higher than imported products across the 9 medicines in the paired analysis (Table 4). Median MPRs of local and imported products were 1.44 and 1.35 respectively, hence patients were paying 7% more for local products.

In an unpaired analysis of all medicines sold to patients in the public sector, locally produced branded generics were 24% lower priced (median MPR = 1.67) than imported branded generics (median MPR = 2.20). For INN generics there was virtually no price difference between imports and products made in Tanzania in the public sector.

#### Public sector patient prices compared to government procurement prices

For the eight locally produced medicines that the government procured and sold to patients in public sector outlets, patients were paying an average of 2.35 times (135% more than) the procurement price (Table 3). For imported products (7 medicines) patients were paying 1.65 times (65% more than) the procurement price.

#### Availability and patient prices in the private sector

The mean availability of imported or locally produced medicines was 82% in the private sector. Availability of local and imported products was 21% and 70%, respectively, with variability for individual medicines. As with the public sector, branded generics were more commonly found than INN generics for local products (19% vs 2%) and imported products (58% vs 12%). No locally produced originator brands were found. The availability of imported originator brands was 7%.

Across 12 paired medicines, patient prices for local and imported products were almost identical at 2.27 and 2.29 times IRP respectively although there was individual variability e.g. imported sulfadoxine/pyrimethamine products (MPR = 10.91) were higher priced than the local products (MPR = 7.27).

Across all medicines, imported branded generics (median MPR = 2.97) were 48% higher priced than those made locally (median MPR = 2.01), as shown in Table 4. Imported INN generics (median MPR = 2.38) were 20% lower priced than imported branded generics, but 15% higher priced than locally produced INN generics (median MPR = 2.07), however, only 12 locally produced INN generics were found. Imported originator brands (median MPR = 8.79) were far higher priced than imported branded generics (median MPR = 2.97) and INN generics (median MPR = 2.38).

#### Summary of results

The findings for Ethiopia and Tanzania show contrasting situations. In Ethiopia, the government paid more overall for locally produced products compared to imports, then applied a lower mark-up on these local products. However, patient prices remained higher for local products compared to imports in public sector outlets. The availability of local products was higher than for imports in both the public and private sectors. In the private sector, patients paid considerably more for imported medicines.

In Tanzania, the government paid less for local products then applied a higher mark-up which resulted in patients paying slightly more for local products compared to imported products in public sector outlets. The availability of local products was lower in public sector outlets compared to imports. In the private sector, imports were far more available than local products and prices were similar.

## Discussion

A key objective of any national medicines policy is to ensure the availability, affordability, and rational use of essential medicines that are safe, effective and quality-assured [12]. Local production is increasingly being considered as a means to improve medicine availability, and improve medicine affordability through lower prices compared to imports.

Substantial manufacturing of medicines takes place in a number of LMICs. India and China are major producers of generic medicines and their role has been critical in meeting public health needs not only in their own countries but also in many other countries, including those in Africa [8, 13]. But African countries are not without local pharmaceutical industries. A 2005 survey found that 37 of 46 African countries in the WHO Africa region possessed some pharmaceutical manufacturing capacity [13]. Since then the number of local manufacturers, their activities and product portfolios, have continued to expand, but not in all African countries [13, 14]. Despite this growth, Africa manufactures less than 2% of the medicines it consumes [15].

Compared to information on medicine prices and availability in general, little is known about the impact of local medicine production on prices and availability. In 2008, Mackintosh and Mujinja surveyed four rural districts in Tanzania and found 46% of selected tracer medicines were made locally, and there were no significant patient price differences between medicines from the three main countries of origin (India, Tanzania and Kenya) [16]. In 2014, Mujinja et al. reported that medicines produced in Tanzania were equally likely to be found in rural and urban areas of the country, but imported medicines displayed an ‘urban bias’ [17]. A further study in Tanzania found a higher proportion of medicines in public sector outlets were made locally (22%) than in the private sector (9%) and the mission sector (12%) [18]. Across all three sectors, 16%, 69% and 15% were made in Tanzania, India and Kenya, respectively. In other countries, three studies found locally produced medicines had lower patient prices compared to imports. Kuanpoth found locally produced ARVs had lower patient prices compared to imported ARVs in Vietnam [19]. Chowdury and Kabir found locally produced over-the-counter essential medicines in Bangladesh had lower patient prices compared to imports [20]. Sweileh et al. found lower patient prices for antibiotics made locally compared to imports [21]. One study, conducted by Shafie and Hassali in Malaysia, found some locally produced generics had higher patient prices compared to imports [22].

To support local producers, some governments have a local preference policy when procuring medicines i.e. they will pay more, up to a fixed percentage, for locally produced medicines than for imports. The World Bank supports this policy, while the Global Fund to Fight AIDS, Tuberculosis and Malaria rejects it [23, 24]. In Tanzania and Ethiopia the local preference policy is 15% and 25%, respectively. In Tanzania, the government was only buying one product per medicine so it was not possible to determine if this 15% local preference policy was being applied. In Ethiopia, for nine of the 25 medicines surveyed, one or more locally made products and one import were purchased, at varying prices and quantities. The reasons for this purchasing practice warrant further study. Perhaps the manufacturers were unable to supply greater quantities, or the government was buying from multiple local manufacturers to provide broader local support. The Ethiopian government’s 25% local preference policy was being exceeded for some medicines. Across the eight medicines where procurement prices were lower for imports compared to locally produced products, annual savings of $3.7 million (from a total budget of $441 million) would result if only the lower-priced imports were purchased.

Interestingly, the Ethiopian government applied a smaller mark-up on higher-priced local products (17%), than on lower-priced imported products (53%). This reduced the difference in patient prices between local and imported products to 22% in public sector outlets. This illustrates another way in which government supports local producers.

The Tanzanian government was paying more for imports compared to local products, but patients in public sector outlets were paying more for local products compared to imports. This was due to the different mark-ups applied by the government (local products 135%, imports 65%). Overall, imports were more available (32%) than local products (21%) in the public sector. The reasons for this should be investigated. What is clear is that in both countries, the government does not buy adequate quantities as medicine availability (local and imports) in the public sector was only 52% in Tanzania and 64% in Ethiopia.

The apparent consumer willingness to pay higher prices for imported products, as seen in the private sector in Ethiopia, may reflect a perception that imports are of higher quality. To boost local industries, the government needs to ensure and publicise the equivalent quality of locally produced products.

Little is known about medicine price components in the private sectors of both countries i.e. manufacturer’s selling prices, mark-ups and other add-ons in the supply chain that make up the final patient price. Local manufacturers may be selling at lower prices but add-ons may significantly increase patient prices making products less affordable for patients. Many WHO/HAI surveys have found it challenging to measure price components [3], so in this survey procurement and selling prices were measured for only one wholesaler per country. This has limited value so is not reported here. However, governments supporting local production should fully investigate price components, including mark-ups, local taxes, rebates and discounts, then regulate markets to ensure their support results in more affordable medicines for patients. South Africa has chosen to use a Single Exit Price (SEP) mechanism that bans discounts and rebates and provides transparent information about the prices of medicines sold in the private sector [25].

Limitations of the methodology include (1) the relatively low number of survey medicines (although over 2500 data points were generated per country) (2) measuring availability only on the survey day (3) not identifying clearance costs for imports purchased by the Ethiopian government (4) not measuring all price components in the pharmaceutical supply chain.

The findings from the survey in Ethiopia are consistent with what is generally understood in some countries in Africa i.e. that imported generics can be lower priced than locally produced products. Initially prices of locally produced medicines may be high, but WHO expects this situation will not remain in the long term in countries working to strengthen their local pharmaceutical industry. In the short to medium term, governments need to develop and implement policies through which they can continue to support local production but, at the same time, prevent high prices being passed on to patients. Different ways of achieving this dual policy objective need to be explored.

In July 2015, two years after this study, Ethiopia launched a ten year strategy and plan of action for pharmaceutical manufacturing [26]. The objectives include improving access to medicines through the local production of quality-assured pharmaceuticals, strengthening the FMHACA, promoting the production of APIs, and creating a research and development platform. As part of the strategy, the government is currently developing an incentive package that supports local manufacturers and ensures patient do not pay through higher prices. This survey establishes a baseline for measuring whether the plan of action results in improved access to medicines through greater availability and lower prices.

## Conclusions

The following are the key conclusions from this study:

- Systems to regularly and reliably monitor the availability and prices of locally produced and imported medicines need to be established to assess the impact of local production on access to medicines.
- Where a survey shows government procurement prices of locally produced products are higher than prices of imports, the procurement prices of *all* medicines should then be reviewed. Local preferences should also be reviewed to ensure medicines are affordable to the population.
- Lower government procurement prices, whether for local or imported products, should be passed on to patients to improve medicine affordability.
- Governments supporting local production need to ensure that where the prices of locally produced medicines are found to be higher than imported ones, they adopt appropriate policies so that high prices are not passed on to patients, as this is contrary to the objective of improving access through local production.
- Supporting local manufacturers through fiscal and/or non-fiscal incentives must be time-bound, developed and implemented in a transparent way, and not paid by patients through higher medicine prices. Balancing local production policies is critically important. Such policies should encourage foreign investments in pharmaceutical manufacturing in developing countries.

Following the two pilot studies that are reported here, the survey tools were refined. The two national reports of all the findings are available on HAI’s website (http://haiweb.org/what-we-do/price-availability-affordability/measuring-the-availability-and-prices-of-locally-produced-and-imported-medicines) [27, 28]. The survey tools (manual and Excel workbook) will be available on the website once finalised. Governments and others who are interested in local production are encouraged to undertake a survey using these tools, then publish reports of the findings on publicly-accessible websites, to increase our understanding of the impact of local production on prices and availability.

## Abbreviations

FMHACA: Food, Medicine and Health Care Administration and Control Authority of Ethiopia
HAI: Health Action International
ICTSD: International Centre for Trade and Sustainable Development
INCO: International Commercial
INN: International Non-proprietary Name
IRP: International Reference Price
LMIC: Low- and Middle-income Country
MPR: Median Price Ratio
NGO: Non-governmental Organisation
TFDA: Tanzanian Food and Drug Administration
UNCTAD: United Nations Conference on Trade and Development
WHO: World Health Organization

## References

[1] World Health Organization (2004). World Medicines Strategy 2004-2007.

[2] World Health Organization/Health Action International. Measuring medicine prices, availability, affordability and price components. 2^nd^ edition. Geneva, 2008. Available at http://haiweb.org/what-we-do/price-availability-affordability/collecting-evidence-on-medicine-prices-availability/. Accessed 8 Nov 2015.

[3] Cameron A, Ewen M, Ross-Degnan D, Ball D, Laing R. Medicine prices, availability, and affordability in 36 developing and middle-income countries: a secondary analysis. Lancet 2009;373:240-249. 10.1016/S0140-6736(08)61762-6 19042012

[4] Van Mourik MSM, Cameron A, Ewen M, Laing RO (2010). Availability, price and affordability of cardiovascular medicines: a comparison across 36 countries using WHO/HAI data. BMC Cardiovasc Disord.

[5] Volman B. Direct costs and availability of diabetes medicines in low- and middle-income countries. WHO/HAI 2008. Available at http://apps.who.int/medicinedocs/en/d/Js18387en/. Accessed 8 Nov 2015.

[6] Cameron A, Bansal A, Dua T, Hill S, Moshe S, Mantel-Teeuwisse A, Saxena S (2012). Mapping the availability, price and affordability of antiepileptic drugs in 46 countries. Epilepsia.

[7] Gelders S, Ewen M, Noguchi N, Laing R. Price, availability and affordability. An international comparison of chronic disease medicines.WHO EMRO/HAI 2006.Available at http://apps.who.int/medicinedocs/documents/s16180e/s16180e.pdf. Accessed 8 Nov 2015.

[8] World Health Organization (2014). Local production for access to medical products. Developing a framework to improve public health.

[9] Kaplan W (2013). Local production and access to medicines in low- and middle-income countries. A literature review and critical analysis.

[10] Kaplan WA, Ritz LS, Vitello M (2011). Local production of medical technologies and its effect on access in low and middle income countries: a systematic review of the literature. South Med Rev.

[11] World Health Organization (2008). World Medicines Strategy 2008-2013.

[12] World Health Organization (2001). How to develop and implement a national drug policy.

[13] Berger M, Murugi J, Buch E et al. Strengthening pharmaceutical innovation in Africa. Council on Health Research for Development (COHRED), New Partnership for Africa’s Development (NEPAD) 2009. Available at http://policycures.org/downloads/COHRED-NEPAD_Strengthening_Pharmaceutical_Innovation_AfricaREPORT.pdf. Accessed 10 Sept 2016.

[14] Mackintosh M, Banda G, Tibandebage P, Wamae W (Eds.).Making medicines in Africa. The political economy of industrializing for local health. International Political Economy Series, 2015. Available at http://www.palgrave.com/us/book/9781137546463. Accessed 10 Sept 2016.

[15] Lopes C. Making medicine in Africa - the untapped possibilities that could save millions of lives. 17 February 2015. Available at http://mgafrica.com/article/2015-02-17-manufacturing-pharmaceuticals-in-africa-the-untapped-opportunity-that-could-save-countless-lives. Accessed 8 Oct 2016.

[16] Mackintosh M, Mujinja PG. Pricing and competition in essential medicines markets: the supply chain to Tanzania and the role of NGOs. IKD Working Paper No. 32. Open University Research Centre on Innovation Knowledge and Development. Available at http://www.open.ac.uk/ikd/sites/www.open.ac.uk.ikd/files/files/working-papers/ikd-working-paper-32.pdf. Accessed 8 Oct 2016.

[17] Mujinja P, Mackintosh M, Justin-Temu M, Wuyts M (2014). Local production of pharmaceuticals in Africa and access to essential medicines: ‘urban bias’ in access to imported medicines in Tanzania and its policy implications. Globalization and Health.

[18] Israel C, Mackintosh M, Tibandebage P, Mhede E, Mujinja PGM. Improving the supply chain for the health sector: what role for local manufacturing? Policy research for development. Working paper 14/6. Available at http://www.repoa.or.tz/documents/REPOA_WP_14_6.pdf. Accessed 8 Oct 2016.

[19] Kuanpoth J (2007). Patents and access to antiretroviral medicines in Vietnam after World Trade Organization accession. J World Intell Prop.

[20] Chowdury N, Kabir ER (2009). Per pill price differences across therapeutic categories: a study of the essential drug brands marketed by multinational and local pharmaceutical companies in Bangladesh. African J Marketing Mgt.

[21] Sweileh W, Jaradat N, Mustafa A (2004). Antibiotic drug cost variations in Palestine: physicians and patients dilemma. An-Najah Univ. J. Res..

[22] Shafie AA, Hassali MA (2008). Price comparison between innovator and generic medicines sold by community pharmacies in the state of Penang, Malaysia. J Generic Med.

[23] The World Bank (2011). Procurement of goods, works, and non-consulting services under IBRD loans and IDA credits and grants by World Bank Borrowers.

[24] Global Fund to Fight AIDS, Tuberculosis and Malaria (2012). Guide to Global Fund policies on procurement and supply management of health products.

[25] Gray AL (2009). Medicine pricing interventions-the South African experience. Southern Med Review.

[26] Ministry of Health and Ministry of Industry, Federal Democratic Republic of Ethiopia. National strategy and plan of action for pharmaceutical manufacturing development in Ethiopia (2015-2025). Developing the pharmaceutical industry and improving access. July 2015. Available at http://www.who.int/phi/publications/Ethiopia_strategy_local_poduction.pdf. Accessed 12 Nov 2015.

[27] World Health Organization/Health Action International. Prices and availability of locally produced and imported medicines in Ethiopia. Available at http://haiweb.org/what-we-do/price-availability-affordabilitymeasuring-the-availability-and-prices-of-locally-produced-and-imported-medicines/ . Accessed 5 Dec 2016.

[28] World Health Organization/Health Action International. Prices and availability of locally produced and imported medicines in Tanzania. Available at http://haiweb.org/what-we-do/price-availability-affordability/measuring-the-availability-and-prices-of-locally-produced-and-imported-medicines. Accessed 5 Dec 2016.

